# APVO210: A Bispecific Anti-CD86-IL-10 Fusion Protein (ADAPTIR™) to Induce Antigen-Specific T Regulatory Type 1 Cells

**DOI:** 10.3389/fimmu.2018.00881

**Published:** 2018-05-25

**Authors:** Laurence Pellerin, Ping Chen, Silvia Gregori, Gabriela Hernandez-Hoyos, Rosa Bacchetta, Maria Grazia Roncarolo

**Affiliations:** ^1^Department of Pediatrics, Division of Stem Cell Transplantation and Regenerative Medicine, Stanford University, Stanford, CA, United States; ^2^Institute for Stem Cell Biology and Regenerative Medicine, Stanford University, Stanford, CA, United States; ^3^San Raffaele Telethon Institute for Gene Therapy (SR-TIGET), IRCCS San Raffaele Scientific Institute, Milan, Italy; ^4^Aptevo Research and Development LLC, Seattle, WA, United States

**Keywords:** IL-10, CD86, T regulatory type 1 cells, tolerogenic dendritic cells, anergy, immunomodulation

## Abstract

IL-10 is a potent immunosuppressive cytokine that promotes the differentiation of tolerogenic dendritic cells (DC-10), and the subsequent induction of antigen-specific T regulatory type 1 (Tr1) cells, which suppress immune responses. However, IL-10 acts on multiple cell types and its effects are not solely inhibitory, therefore, limiting its use as immunomodulant. APVO210 is a bispecific fusion protein composed of an anti-CD86 antibody fused with monomeric IL-10 (ADAPTIR™ from Aptevo Therapeutics). APVO210 specifically induces IL-10R signaling in CD86^+^ antigen-presenting cells, but not in T and B cells. In this study, we tested whether APVO210 promotes the differentiation of tolerogenic DC-10 and the differentiation of antigen-specific CD4^+^ Tr1 cells *in vitro*. We compared the effect of APVO210 with that of recombinant human (rh) IL-10 on the *in vitro* differentiation of DC-10, induction of alloantigen-specific anergic CD4^+^ T cells, enrichment in CD49b^+^LAG3^+^ Tr1 cells mediating antigen-specific suppression, and stability upon exposure to inflammatory cytokines. APVO210 induced the differentiation of tolerogenic DC (DC-A210) that produced high levels of IL-10, expressed CD86, HLA-G, and intermediate levels of CD14 and CD16. These DC-A210 induced alloantigen-specific anergic T-cell cultures (T-alloA210) that were enriched in CD49b^+^ LAG3^+^ Tr1 cells, produced high levels of IL-10, and had suppressive properties. The phenotype and high IL-10 production by DC-A210, and the alloantigen-specific anergy of T-alloA210 were preserved upon exposure to the inflammatory cytokines IL-1β, IL-6, and TNF-α. The effects of APVO210 were comparable to that of dimeric rh IL-10. In conclusion, our data demonstrate that APVO210 drives the differentiation of tolerogenic DC and functional alloantigen-specific Tr1 cells *in vitro*. Since APVO210 specifically targets CD86^+^ cells, we hypothesize that it will specifically target CD86^+^ DC to induce Tr1 cells *in vivo*, and mediate antigen-specific immunological tolerance by induction of tolerogenic DC and Tr1 cells.

## Introduction

IL-10 is an immunomodulatory cytokine that has pleiotropic roles. The inhibitory roles of IL-10 directly consist in dampening CD4^+^ T-cell proliferation, in inhibiting production of inflammatory cytokines by CD4^+^ T cells, such as TNF-α, or by antigen-presenting cells (APC), such as IL-1 and TNF-α (1–4). In addition, IL-10 downregulates the antigen-presenting capacity of APC by downregulating the expression of costimulatory molecules, the production of proinflammatory cytokines, the expression of class II MHC and, therefore, indirectly reducing their ability to induce activation and proliferation in T cells (5, 6). However, IL-10 is also a potent growth and differentiation factor for activated human B lymphocytes (7, 8), and, in conjunction with low doses of IL-2, can favor rather than inhibit the proliferation of activated CD8^+^ T cells (9, 10). Because of its immunosuppressive functions, *in vivo* administration of IL-10 has been tested in murine models of immune-mediated diseases, including inflammatory bowel disease (IBD), rheumatoid arthritis (RA), and type 1 diabetes, and proved to alleviate the inflammation and the undesired immune response [Reviewed in Ref. (11–14)]. Therefore, clinical trials were conducted to harness the immunosuppressive activity of IL-10 (11, 14). However, in phase I trials to treat RA, serial administrations of IL-10 had limited efficacy and induced clinical complications, such as neutrophilia, monocytosis, and lymphopenia (15). IL-10 therapy was also tested in phase II clinical trials in IBD and psoriasis. Systemic administration of IL-10 could not improve IBD symptoms or prevent reoccurence of the disease (16–18), while subcutaneous injections of IL-10, below the psoriatic plaques, could decrease the dermal lymphocyte infiltrates and ameliorate the clinical symptoms (19–22). Thus, psoriasis remains the only example of disease where IL-10 therapy showed efficacy to control undesired immune reactions, most likely because IL-10 was injected at the site of inflammation, and could exert its immunosuppressive function locally.

IL-10 is essential for the induction of T regulatory type 1 (Tr1) cells, a subset of T regulatory cells (Treg) dedicated to the maintenance of peripheral immune tolerance. FOXP3^+^ Treg and Tr1 cells are the best described subsets of Tregs with independent lineage origins, but similar mechanisms of action (23–26). Tr1 cells were identified based on their high IL-10 production (27, 28), and later characterized by the expression of the surface molecules CD49b and LAG3 (29). Tr1 cells also secrete TGF-β and variable levels of IFN-γ, but not IL-2, IL-4, or IL-17 (27, 28, 30), they are anergic (hyporesponsive upon secondary antigen stimulation) and suppress antigen-specific CD4^+^ T-cell responses (26, 31). In addition to its role in Tr1 differentiation and suppressive function, IL-10 is also essential for the differentiation of tolerogenic dendritic cells (DC-10). DC-10 are potent inducers of antigen-specific Tr1 cells *in vitro*, are present *in vivo*, and mediate immunological tolerance as suggested, for example, in the maintenance of feto-maternal tolerance (32–36). Our group and others have clearly demonstrated the crucial role of Tr1 cells for the establishment and maintenance of immune tolerance in hematopoietic stem cell transplantation (HSCT) (26, 27, 30, 37–39), celiac disease (40), and allergic diseases (41). Therefore, the *in vivo* use of IL-10 producing Tr1 cells has been explored with the rationale that antigen-specific Tr1 cells would exert their suppressive and antiinflammatory effects without causing general immunosuppression. The *in vivo* efficacy of Tr1 cells has been showed in murine models of inflammatory diseases (28, 29, 42) of MHC mismatched bone marrow (43, 44) and of solid organ transplantation (45, 46). Furthermore, clinical trials exploring antigen-specific Tr1 cells as a cell therapy in Crohn’s disease (47), and in HSCT to prevent graft versus host disease (GvHD) have been performed (48) or are ongoing (ClinicalTrials.gov Identifier: NCT03198234). These trials have shown the safety of using Tr1 cells *in vivo*, supporting that the local delivery of IL-10 at the site of APC–T cell interaction does not lead to the development of adverse effects.

An alternative way to control autoreactive and alloreactive T-cell responses is through direct modulation of APC function. Control of CD4^+^ and CD8^+^ T-cell activation by APC requires interaction with costimulatory molecules that can either promote or inhibit T-cell effector function and expansion (49). In particular, CD80 and CD86 expressed by APC bind to the costimulatory molecule CD28 or the inhibitory molecule CTLA4 on T cells (50). In addition, absence of costimulatory signals during TCR-mediated activation results in T-cell anergy (51, 52). Modulating co-stimulatory pathways is, therefore, of great interest to dampen T-cell responses associated with autoimmune diseases and GvHD (53, 54).

APVO210 is an ADAPTIR™ (modular protein technology) molecule developed by Aptevo Therapeuthics that contains a blocking anti-CD86 single-chain Fv coupled to an engineered monomeric form of the human IL-10 (55). The central portion of the protein is an engineered immunoglobulin Fc domain that provides extended *in vivo* half-life and lacks effector function. Monomeric IL-10 induces lower IL-10R signaling compared to dimeric human IL-10. Therefore, APVO210 is able to target and block the co-stimulatory receptor CD86 on APC, while selectively triggering IL-10 receptor signaling on those cells. Indeed, APVO210 induces STAT3 phosphorylation on monocytes and DC, but not on resting or activated T or B cells *in vitro*. APVO210 has a longer half-life (approximately 40 h; Hernandez-Hoyos et al., unpublished data) compared to IL-10 [1 h (56)]. Furthermore, APVO210 is more potent than anti-CD86 mAb or dimeric soluble IL-10 to block the expansion of human peripheral blood mononuclear cells (PBMC) in a mouse model of xeno-GvHD.^1^ Thus, APVO210 displays a synergic activity between IL-10 and the anti-CD86 mAb as compared to each of those molecules alone (see text footnote 1).

Based on these data, we hypothesized that APVO210 could be a strong and selective immunomodulant by inducing tolerogenic properties in CD86^+^ target cells that will in turn affect T-cell responses and favor Tr1 cell differentiation.

Our results show that DC differentiated *in vitro* in the presence of APVO210 (DC-A210) express intermediate levels of CD14 and CD16, high levels of CD86 and HLA-G, and produce high levels of IL-10. In addition, T cells differentiated with DC-A210 (T-alloA210) present alloantigen-specific anergy, comprise a significant population of CD49b^+^LAG3^+^ Tr1 cells, are highly suppressive and produce IL-10. The phenotype and functional properties of DC-A210 and the anergy of T-cell cultures stimulated by these DC remained stable upon exposure to inflammatory cytokines, and were comparable to that of the DC-10 and T-cell cultures generated with DC-10 in the presence of rhIL-10. Overall, these findings support the potent immunomodulatory function of APVO210 as a molecule able to drive the induction of tolerogenic DC and Tr1 cells that could be exploited *in vivo*.

## Materials and Methods

### Study Subjects

Peripheral blood cells (buffy coats) of healthy subjects were purchased from the Stanford University Blood Center (Palo Alto, CA, USA) and, therefore, exempt consent for the study. PBMCs were isolated by Ficoll–Paque density centrifugation using Ficoll–Paque plus (GE Healthcare, Chicago, IL, USA).

### Dendritic Cell Differentiation

DCs were differentiated from CD14^+^ monocytes that were isolated from PBMC using CD14^+^ microbeads (Miltenyi Biotec, San Diego, CA, USA) according to the manufacturer’s instructions. CD14^+^ monocytes were cultured for 7 days in RPMI 1640 (Life Technologies, Carlsbad, CA, USA) supplemented with 10% pooled AB human serum (HS; Sigma-Aldrich, Saint Louis, MO, USA) and 100 U/mL penicillin/streptomycin (Life Technologies, Carlsbad, CA, USA) in the presence of 10 ng/mL of recombinant human (rh) IL-4 and 100 ng/mL of rhGM-CSF (R&D Systems, Minneapolis, MN, USA), with the addition of 10 ng/mL (or 0.27 nM) of rhIL-10 (BD Biosciences, San Jose, CA, USA) to differentiate DC-10, or with the addition of 1 or 10 nM of APVO210 to generate DC-A210 cells (Figure S1 in Supplementary Material). Cells cultured in the presence of rhIL-4 and rhGM-CSF only were matured with additional 5 μg/mL of monophosphoryl lipid A (MPLA; InvivoGen, San Diego, CA, USA) for the last 2 days of cell culture to generate control mature DC (mDC). For each experiment, the cells were phenotypically analyzed, or irradiated and used to stimulate allogeneic T cells. To test the stability of phenotype and function of the cells upon exposure to inflammatory cytokines, DC-A210 and DC-10 were plated in the presence or absence of 5 ng/mL of rhIL-1β and rhTNF-α (R&D Systems, Minneapolis, MN, USA). After 24 h, the cells were collected and expression of CD14 and CD16 was evaluated by flow cytometry. To measure cytokine production by DC-A210 and DC-10, the cells were stimulated for 48 h with 200 ng/mL of LPS in the presence or absence of 5 ng/mL of rhIL-1β and rhTNF-α. Supernatants were collected, and production of IL-6 and IL-10 was measured by ELISA.

### Tr1 Cell Induction

CD4^+^ T cells were isolated from PBMC by positive selection using CD4 microbeads (Miltenyi Biotec, San Diego, CA, USA) according to the manufacturer’s instructions. DC-A210, DC-10, or mDC were co-incubated with allogeneic CD4^+^ T cells at 1:10 DC:CD4^+^ T-cell ratio in X-VIVO 15 medium supplemented with gentamycin (Lonza, Switzerland) and 5% HS (complete medium; Figure S1 in Supplementary Material). When indicated, APVO210 was added at a concentration of 1 or 10 nM at day 0 and day 5 to the DC-A210–CD4^+^ T-cell co-culture (T-alloA210 1 or 10 nM, respectively). rhIL-10 was added at a concentration of 10 ng/mL at day 0 and 5 of the DC-10–CD4^+^ T-cell co-culture (T-allo10). Control CD4^+^ T cells stimulated with mDC are referred to as T-allo cells.

### DC and T-Cell Phenotype by Flow Cytometry

Cells were incubated for 10 min with a FcR blocking reagent (Miltenyi Biotec, San Diego, CA, USA). DC were stained for 30 min at 4°C with anti-CD14 (eBioscience, San Diego, CA, USA), anti-CD16 and anti-CD86 (BioLegend, San Diego, CA, USA). T cells were stained for 15 min at 37°C with anti-CD3, anti-CD4, anti-CD45RA (BioLegend, San Diego, CA, USA), Live/Dead Fixable Aqua Dead Cell Stain Kit (L/D Aqua; Life Technologies, Foster City, CA, USA), anti-CD49b and anti-LAG3 (Miltenyi Biotec, San Diego, CA, USA). Data were acquired using a FACSAria II (BD Biosciences, San Jose, CA, USA) and analyzed with FlowJo 9.8.3 software (FlowJo LLC, Ashland, OR, USA).

### Cytokine Determination by ELISA

DC-A210, DC-10, and mDC were left unstimulated or stimulated with 200 ng/mL of lipopolysaccharide (LPS; Sigma-Aldrich, Saint Louis, MO, USA) for 48 h. Supernatants were collected, and concentrations of IL-6, IL-10, and TNF-α were measured by ELISA according to the manufacturer’s instructions (BD Bioscience, San Jose, CA, USA). T-alloA210, T-allo10, and T-allo cells were left unstimulated or stimulated with allogeneic mDC (allo-mDC) for 48 h (at a 1:10 DC:T-cell ratio), supernatants were collected, and concentrations of IL-4, IL-10, and IFN-γ were measured by ELISA.

### IL-10 Determination by Quantitative RT-PCR (qRT-PCR)

DCs were left unstimulated or stimulated with 200 ng/mL of LPS (Sigma-Aldrich, Saint Louis, MO, USA) for 24 h. Total RNA was extracted with the RNeasy Plus Mini kit (Qiagen, Valencia, CA, USA) and was used as template to synthesize cDNA for qRT-PCR analysis using a QuantStudio™ 7 Flex Real-Time PCR System (Applied Biosystems, Foster City, CA, USA). qRT-PCR was performed in triplicate wells using the TaqMan^®^ Fast Universal PCR Master Mix (Applied Biosystems, Foster City, CA, USA). Relative levels of gene expression among samples were determined by using the ΔΔ cycle threshold method. Protein Lateral Stalk Subunit P0 (RPLP0) gene expression was used for normalization.

### Cytokine Determination by Capture Assay

T-alloA210, T-allo10, and T-allo cells were left unstimulated or stimulated with 50 ng/mL of phorbol 12-myristate 13-acetate (PMA; Sigma-Aldrich, Saint Louis, MO, USA) and 1 µg/mL of ionomycin (Sigma-Aldrich, Saint Louis, MO, USA) for 6 h. The optimal length of stimulation to detect IL-10 secretion was determined by a time course experiment (data not show). A secretion assay–detection kit (Miltenyi Biotec, Bergisch Gladbach, Germany) was used according to the manufacturer’s instructions to simultaneously detect IL-10 and IFN-γ production. The cells were concomitantly stained with anti-CD3 and anti-CD4 antibodies, and analyzed by flow cytometry.

### T-Cell Proliferation Assay

To assess the proliferative response to a secondary allogenic stimulation, T-alloA210, T-allo10, and T-allo cells were labeled with CellTrace™ carboxyfluorescein succinimidyl ester (CFSE; Life Technologies, Carlsbad, CA, USA), and plated with allogenic mDC (from the same donor used during the primary stimulation) at a 10:1 T:DC ratio, in 200 µL of complete medium in 96-well round bottom plates. To test if T-alloA210 cells were able to respond to a polyclonal stimulation, the cells were stimulated with Dynabeads coated with anti-CD3 and anti-CD28 antibodies (Life Technologies, Carlsbad, CA, USA) at a 1:20 bead:cell ratio. After 72 h, the percentage of CFSE-low divided cells within the CD3^+^CD4^+^ population was assessed by flow cytometry. Percentage of T-cell anergy was calculated using the formula: “(% proliferation T-allo with mDC – % proliferation T-alloA210 or T-allo10 with mDC)/% proliferation T-allo with mDC.” Stability of T-alloA210 and T-allo10 cell anergy was tested in the presence or absence of 5 ng/mL of rhIL-1β, rhTNF-α, and rhIL-6 (R&D Systems, Minneapolis, MN, USA) upon 72 h stimulation by allogenic mDC.

### T-Cell Suppression Assay

To evaluate the suppressive activity of T-alloA210 and T-allo10 cells, autologous CD4^+^ T cells (responder cells “R”) were stained with CellTrace™ Violet (Life Technologies, Carlsbad, CA, USA), activated with allo-mDC (from the same donor used during the primary stimulation), and CFSE-stained T-alloA210 or T-allo10 cells (suppressor cells “S”) were added at a 1:10:10 mDC:R:S ratio, in 200 µL of complete medium in 96-well round bottom plate. The percentage of Violet low divided responder cells within the CD3^+^CD4^+^ population was assessed by flow cytometry after 4 or 5 days. Percentage of suppression was calculated using the formula: “(% proliferation R – % proliferation R with S)/% proliferation R.”

### Statistical Analyses

Statistical analyses were performed using Graphpad Prism 6.07 (GraphPad Software, Inc., La Jolla, CA, USA). Results are presented as median percentage of positive cells ± (range), median anergy ± (range), or median concentration ± (range). Wilcoxon test and paired *t*-tests were used to determine the statistical significance of the data.

## Results

### DC Differentiated in the Presence of APVO210 Have a Tolerogenic Phenotype and Function

**Figure 1:**
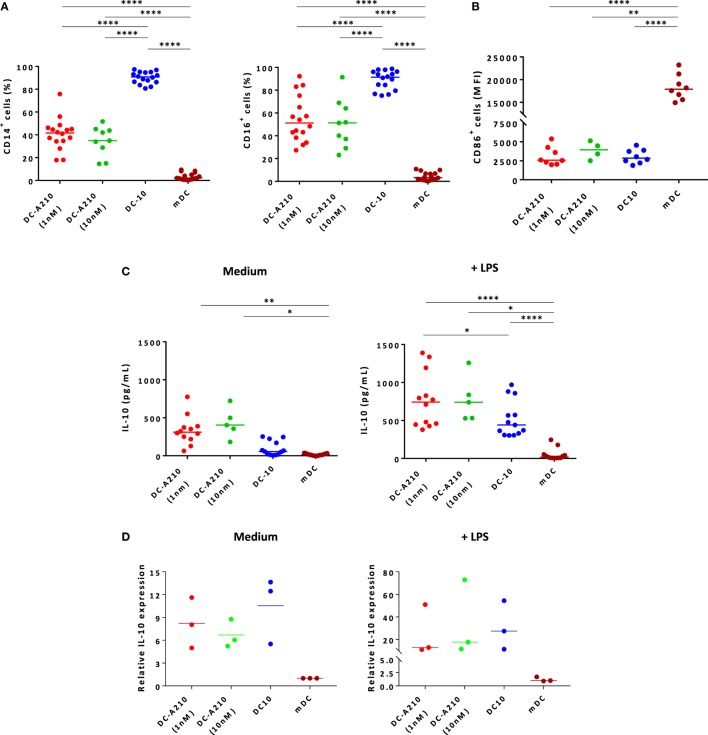
Tolerogenic DC can be differentiated from CD14^+^ monocytes in the presence of APVO210. DC were differentiated from CD14^+^ monocytes in the presence of IL-4 + GM-CSF [mature DC (mDC)], and of exogenous rhIL-10 [dendritic cells (DC-10)], or of APVO210 used at two different concentrations (DC-A210, 1 or 10 nM). **(A)** Percentage of CD14^+^ and CD16^+^ was assessed by flow cytometry in DC-A210 (1 nM: *n* = 16; 10 nM: *n* = 9), DC-10 (*n* = 16) and mDC (*n* = 16). **(B)** Median fluorescence intensity of CD86 is shown in CD86^+^ in DC-A210 (1 nM: *n* = 7; 10 nM: *n* = 3), DC-10 (*n* = 8) and mDC (*n* = 8). **(C)** Production of IL-10 was evaluated by ELISA in culture supernatants of DC-A210 (1 nM, *n* = 12; 10 nM, *n* = 5), DC-10 (*n* = 13), and mDC (*n* = 13) after 48 h, in the presence or absence of LPS stimulation. **(D)** *IL-10* mRNA expression levels were evaluated in DC-A210, DC-10, and mDC (*n* = 3) by quantitative RT-PCR after 24 h in the presence or absence of LPS stimulation. Data are represented in fold-change of expression compared to mDC. Median values are indicated and each dot represents a single donor. Paired *t*-tests with Bonferroni correction were performed for statistical analysis. *p < 0.05, **p < 0.005, ****p < 0.0001.

We characterized the phenotype and cytokine production of monocyte-derived DC differentiated in the presence of 1 or 10 nM of APVO210 (DC-A210), and compared them to monocyte-derived DC differentiated in the presence of rhIL-10 at 10 ng/mL or 0.27 nM (DC-10, Figure 1; Figures S1 and S2 in Supplementary Material). The percentage of DC-A210 expressing CD14 and CD16 (Figure 1A; Figure S2A in Supplementary Material) was significantly lower compared to that of DC-10 (*p* < 0.0001), but significantly higher compared to that of monocyte-derived mDC (*p* < 0.0001). The percentage of CD86^+^ cells was high and comparable in DC-A210, DC-10, and mDC cultures (Figure S2A in Supplementary Material). The median fluorescence intensity of CD86 was comparable in DC-A210 and DC-10, but lower compared to that in mDC (Figure 1B; *p* < 0.0001). In addition, HLA-G expression was high and comparable in DC-A210 [92.3% (80.1–100%), *n* = 4] and DC-10 [91.2% (80.9–100%), *n* = 4; data not shown]. No phenotypical differences were observed when APVO210 was added at 1 or 10 nM.

DC-A210 spontaneously secreted significantly higher levels of IL-10 compared to DC-10 (*p* = 0.0066) and mDC (*p* = 0.0006; Figure 1C). They secreted negligible amounts of TNF-α and low or undetectable amounts of IL-6 (Figure S2B in Supplementary Material). Upon activation with LPS, DC-A210 secreted significantly higher levels of IL-10 compared to DC-10 (*p* < 0.05) and mDC (*p* < 0.0001; Figure 1C). DC-A210 cultured with 1 nM of the compound, but not with 10 nM, secreted significantly higher levels of TNF-α compared to DC-10 (*p* < 0.05 at a concentration of APVO210 of 1 nM, and *p* < 0.05 at a concentration of APVO210 of 10 nM) and levels of IL-6 that were comparable to DC-10, but significantly higher than those of mDC (*p* < 0.005; Figure S2B in Supplementary Material). To ensure that we detected *de novo* IL-10 secretion by DC-A210 and not IL-10 released from the APVO210 molecule, we performed qRT-PCR to evaluate *IL-10* mRNA levels in DC-A210, DC-10, and mDC. Both DC-A210 and DC-10 expressed higher levels of *IL-10* mRNA compared to mDC (Figure 1D). There was no statistical difference in the levels of *IL-10* mRNA detected in DC-A210 compared to DC-10, regardless of whether or not they were stimulated (Figure 1D). Overall, these data indicate that APVO210 allows the differentiation of tolerogenic DC that present an intermediate phenotype between DC-10 and mDC, and produce high levels of IL-10.

### DC-A210 Promote the Differentiation of Antigen-Specific Tr1 Cells

**Figure 2:**
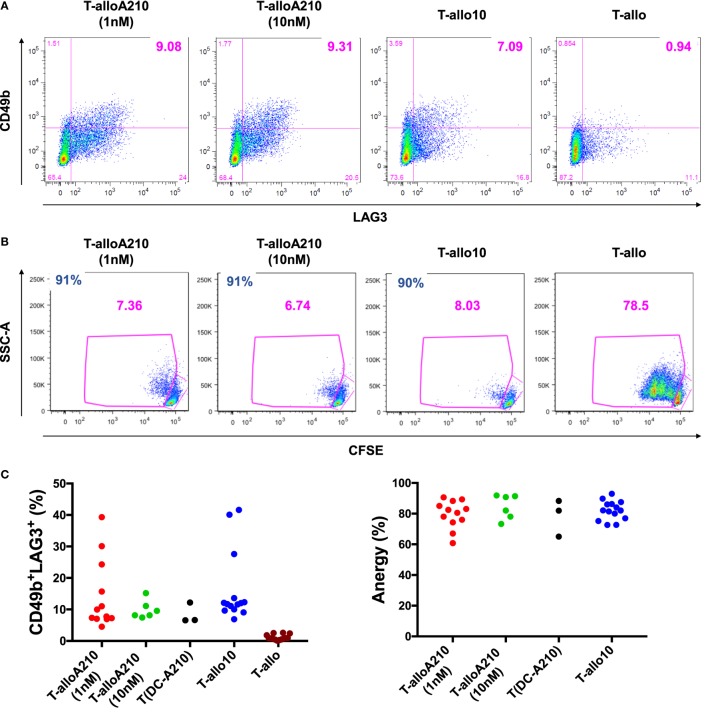
DC-A210 induce alloantigen-specific T regulatory type 1 (Tr1) cells *in vitro*. DC differentiated in the presence of APVO210 (DC-A210), DC-10, and mature DC (mDC) were incubated for 10 days with allogenic CD4^+^ T cells. **(A)** The presence of CD49b^+^LAG3^+^ Tr1 cells was tested by flow cytometry in T cells cultured with allogenic DC-A210 + APVO210 (T-alloA210: 1 nM, *n* = 12 or 10 nM, *n* = 6), with DC-A210 with no addition of APVO210 [T(DC-A210)], with DC-10 + rhIL-10 (T-allo10, *n* = 14) and with mDC (T-allo, *n* = 14). Data are shown for a representative donor. **(B)** Proliferation of T-alloA210, T-allo10, and T-allo cells stained with CFSE and stimulated for 3 days with allogeneic mDC was evaluated by means of CFSE dye dilution using flow cytometry. Percentage of anergy calculated using the formula “(% proliferation T-allo with mDC − % proliferation T-alloA210 or T-allo10 with mDC)/% proliferation T-allo with mDC.” Data of one representative donor are shown, percentage of anergy is indicated in blue. **(C)** Separate plots for phenotype and anergy are shown. Wilcoxon tests with Bonferroni correction were performed for statistical analysis.

We next tested the capacity of DC-A210 to induce alloantigen-specific CD49b^+^LAG3^+^ Tr1 cells from CD4^+^ T cells in the presence of additional APVO210 (T-alloA210). As controls, T-allo10 and T-allo cells were differentiated with either DC-10 in the presence of exogenous rhIL-10, or mDC from the same allogenic donor, respectively (Figure S1 in Supplementary Material). After 10 days, the previously established optimal time of exposure to rhIL-10 (29, 57), the percentage of CD49b^+^LAG3^+^ Tr1 cells was comparable in T-alloA210 [8.9 (4.5–39.3)] and in T-allo10 cell cultures [11.9 (6.9–41.6); Figures 2A,C; Table 1], whereas, as expected, CD49b^+^LAG3^+^ Tr1 cells were not detectable in the control T-allo cell cultures (Figure 2A; Table 1). We did not observe a significant difference in the percentage of CD49b^+^LAG3^+^ Tr1 cells in T-alloA210 cells induced with DC-A210 (1 nM) and DC-A210 (10 nM).

**Table 1:** Percentage^a^ of CD49b^+^LAG3^+^ and anergy in T cells.

Culture conditions^b^	CD49b^+^LAG3^+^	Anergy

	%	
T-allo (*n* = 14)	0.9 (0.1–2.6)	/
T-allo10 (*n* = 14)	11.9 (6.9–41.6)	82.0 (72.6–93.0)
T-alloA210 (1 nM, *n* = 12)	8.9 (4.5–39.3)	81.5 (60.8–90.6)
T-alloA210 (10 nM, *n* = 6)	8.9 (7.4–15.2)	86.4 (73.3–92.0)
T(DC-A210) (1 nM, *n* = 3)	6.63 (6.6–12.2)	82.0 (65.0–88.3)

*^a^Values represent median of different experiments with (ranges)*.

*^b^T-allo = CD4^+^ T cells induced by mature DC; T-allo10 = CD4^+^ T cells induced by DC-10 in the presence of rhIL-10; T-alloA210 = CD4^+^ T cells induced by DC-A210 in the presence of APVO210; and T(DC-A210) = CD4^+^ T cells induced by DC-A210 with no further addition of APVO210*.

T-alloA210 cells presented high alloantigen-specific anergy [81.5 (60.8–90.6)] that was comparable to the anergy of T-allo10 cells [82 (72.6–92.99); Figures 2B,C; Table 1], when re-stimulated with allo-mDC from the same donor used during the induction of T-alloA210 cells. As expected, T-alloA210, T-allo10, and T-allo cell cultures strongly proliferated in response to either polyclonal stimulation or third party mDC stimulation (Figure S3 in Supplementary Material and data not shown). We previously described that culture of CD4^+^ T cells with DC-10 with no addition of exogenous IL-10 is sufficient to induce alloantigen-specific anergic cell cultures that contain CD49b^+^LAG3^+^ Tr1 cells (32, 58). Therefore, we assessed whether addition of APVO210 during the DC-A210-T-cell co-culture was dispensable for differentiation of alloantigen-specific Tr1 cells. Our results showed that the sole presence of DC-A210 (1 nM) was sufficient to induce anergic T-alloA210 that contained CD49b^+^LAG3^+^ Tr1 cells (Figure 2C; Table 1). We did not observe a significant difference in the anergy of T-alloA210 cells induced with DC-A210 (1 nM) and DC-A210 (10 nM). Overall, our data showed that DC-A210 alone or in culture with APVO210 are as potent as DC-10 to induce antigen-specific anergy and differentiation of antigen-specific CD49b^+^LAG3^+^ Tr1 cells.

### T-alloA210 Cells Produce IL-10 and Suppress Autologous Primary Proliferative Responses

**Figure 3:**
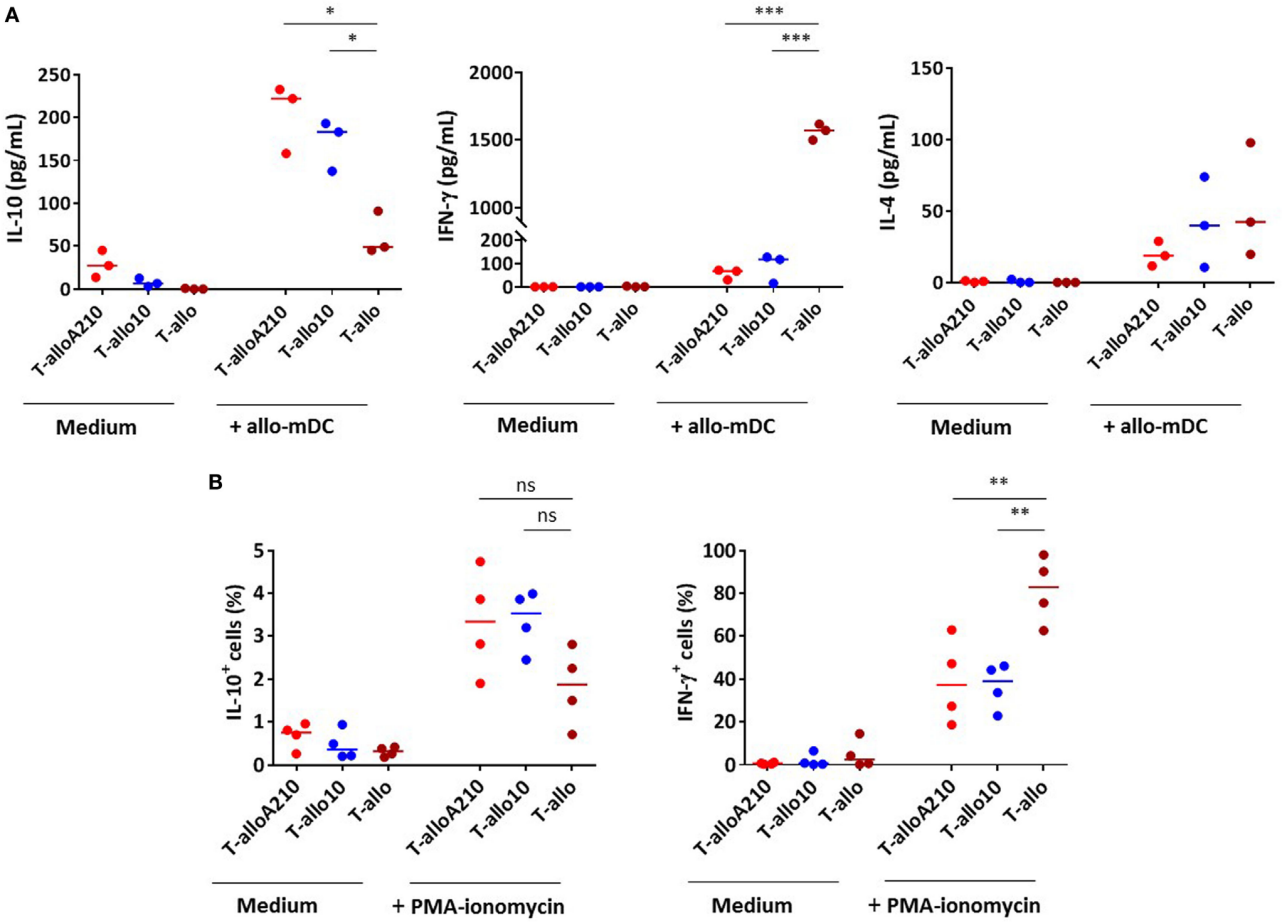
T-alloA210 and T-allo10 cells exhibit a similar cytokine production profile. DC differentiated in the presence of APVO210 (DC-A210), DC-10, and mature DC (mDC) were incubated for 10 days with allogenic T cells. T cells were cultured with allogenic DC-A210 + APVO210 (T-alloA210: 1 or 10 nM), with DC-10 + IL-10 (T-allo10) or with mDC (T-allo). **(A)** Levels of IL-10, IFN-γ, and IL-4 were evaluated by ELISA in culture supernatants after 48 h of stimulation with allogenic mDC (data are expressed in pg/mL). **(B)** Percentages of cells producing IL-10 and IFN-γ were evaluated by flow cytometry, using a secretion capture assay, after 6 h of stimulation with PMA and ionomycin. Median values are indicated, each dot represents a single donor. Paired *t*-tests with Bonferroni correction were performed for statistical analysis. *p < 0.05, **p < 0.001, ***p < 0.0001.

IL-10 production and suppressive activity are essential immunomodulatory properties of T-allo10 cells (26, 32, 33). Therefore, we tested the ability of alloantigen-specific T-alloA210 cell cultures to secrete IL-10 and suppress autologous T-cell proliferation. Upon secondary stimulation with allogenic mDC, T-alloA210 cells secreted predominantly IL-10, and low levels of IL-4 (Figure 3A). The levels of IL-10 produced by T-alloA210 cells were comparable to that of T-allo10 cells and significantly higher compared to those produced by control T-allo cell cultures (*p* < 0.05). Moreover, levels of IFN-γ secreted by T-alloA210 and T-allo10 cells in response to allogenic mDC stimulation were low and comparable, and significantly lower than those produced by T-allo cells (*p* < 0.0001; Figure 3A). The presence of IL-10-producing cells in the T-alloA210 and T-allo10 cells was confirmed by secretion capture assay upon polyclonal stimulation (Figure 3B; Figure S4 in Supplementary Material). The percentages of IL-10^+^ and IFN-γ^+^ cells were comparable in T-alloA210 and T-allo10 cultures, with a lower percentage of cells producing IFN-γ as compared to the control T-allo cell cultures (*p* < 0.001). Notably, the IL-10^+^ cells detected in this assay were also IFN-γ^+^ (Figure S4 in Supplementary Material).

**Figure 4:**
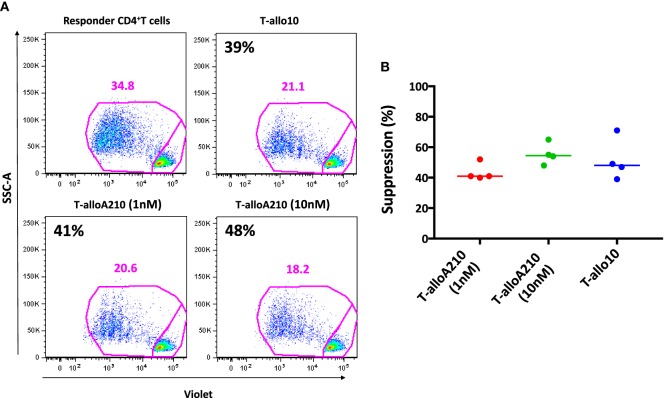
T-alloA210 cells suppress proliferation of autologous CD4^+^ T cells. DC differentiated in the presence of APVO210 (DC-A210), DC-10, and mature DC (mDC) were incubated for 10 days with allogenic T cells. Suppressor T cells (“S”) cultured with allogenic DC-A210 + APVO210 (T-alloA210: 1 or 10 nM) and DC-10 + IL-10 (T-allo10; *n* = 4) were tested for their ability to suppress proliferation of autologous responder CD4^+^ T (“R”) cells in response to allogenic mDC stimulation. Responder T cells were stained with Violet dye, and their proliferation was evaluated by flow cytometry. Percentage of suppression was calculated using the formula “(% proliferation R − % proliferation R with S)/% proliferation R.” **(A)** Data of one representative donor are shown; percentage of suppression is indicated in black. **(B)** Plot showing cumulative data of suppression. Median values are indicated, each dot represents a single donor, and lines indicate median values. Paired *t*-tests with Bonferroni correction were performed for statistical analysis.

T-alloA210 cells were tested for their ability to suppress the proliferative responses of autologous CD4^+^ T cells activated with allogenic mDC from the same donor used during the induction of T-alloA210 cells. The suppressive capacity of T-alloA210 cells was comparable to that of T-allo10 cells [Figures 4A,B; percentage of suppression T-alloA210 1 nM: 41% ± (40–52), T-alloA210 10 nM: 55% ± (48–65), and T-allo10: 48% (39–71)]. Overall, our data show that T-alloA210, similarly to T-allo10 cells, produce high levels of IL-10 and are able to suppress the proliferation of autologous CD4^+^ T cells.

### DC-A210 and T-alloA210 Cells Are Stable in Inflammatory Conditions

**Figure 5:**
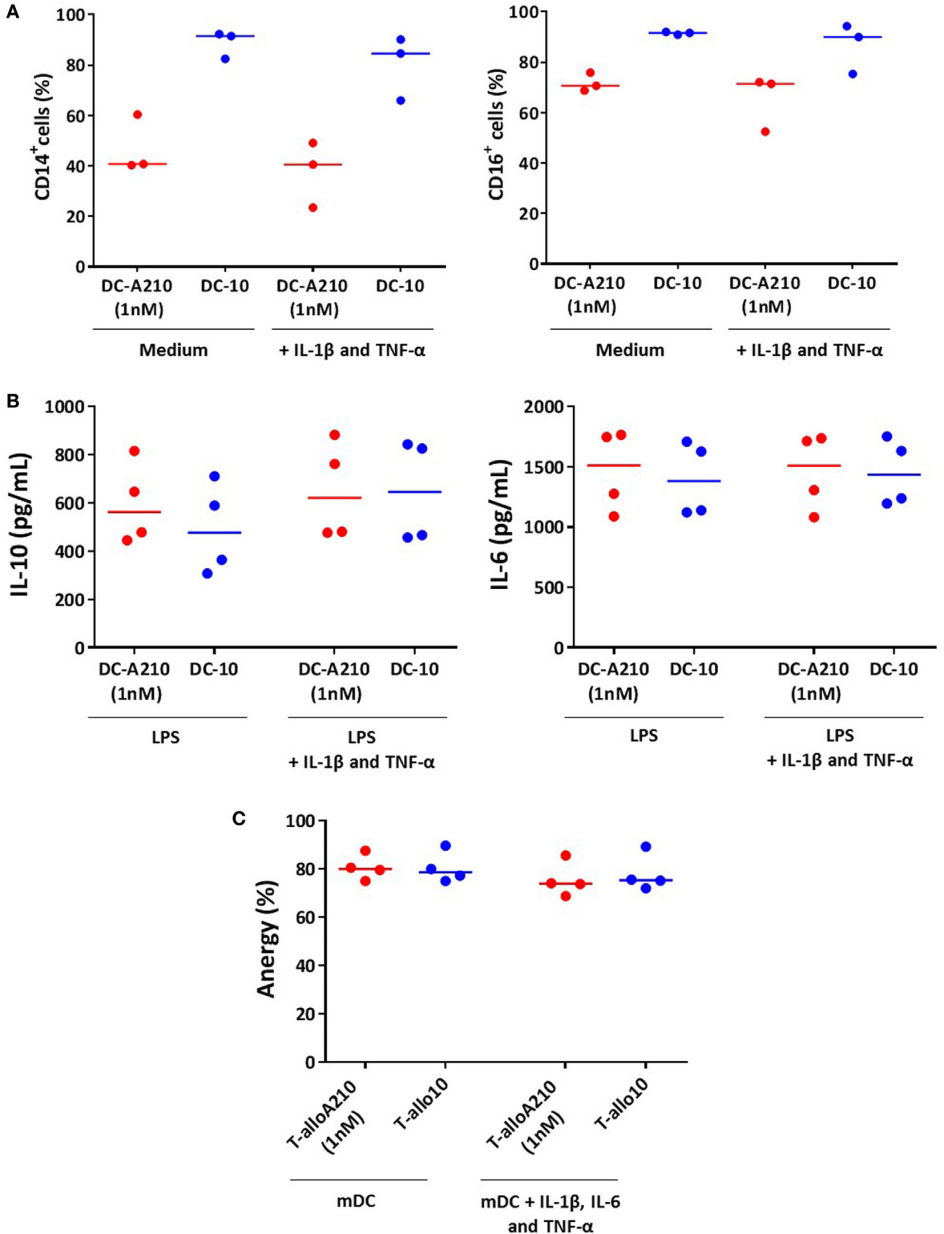
DC-A210 and T-alloA210 cells are stable upon exposure to inflammatory cytokines *in vitro*. **(A)** DC differentiated in the presence of APVO210 (DC-A210) and tolerogenic DC (DC-10) were incubated for 24 h with IL-1β and TNF-α, and percentage of CD14^+^, and CD16^+^ cells was evaluated by flow cytometry (*n* = 3). **(B)** DC-A210 and DC-10 were stimulated with LPS for 48 h in the presence or absence of IL-1β and TNF-α, and production of IL-10 and IL-6 was evaluated by ELISA in culture supernatants (*n* = 4). **(C)** T cells were cultured with allogenic DC-A210 + APVO210 (T-alloA210: 1 nM), DC-10 + IL-10 (T-allo10) or mature DC (mDC) (T-allo). T-alloA210, T-allo10, and T-allo were stained with CFSE, stimulated for 3 days with allogeneic mDC in the presence or absence of IL-1β, IL-6, and TNF-α, and proliferation was evaluated by means of CFSE dye dilution using flow cytometry (*n* = 4). Percentage of anergy calculated using the formula (% proliferation (T-allo + mDC) − % proliferation (T-alloA210 or T-allo10 + mDC)/% proliferation of T-allo + mDC) is shown. Median values are indicated, each dot represents a donor, and lines indicate median values. Paired *t*-tests with Bonferroni correction were performed for statistical analysis.

We next investigated whether the phenotype and cytokine production of DC-A210, and the functional anergy of T-alloA210 cells were stable upon *in vitro* exposure to the inflammatory cytokines IL-1β, IL-6, and TNF-α. The expression of CD14 and CD16 in DC-A210 and DC-10 did not change after 24 h exposure to IL-1β and TNF-α (Figure 5A). In addition, the cytokine production profile of DC-A210 and DC-10 stimulated with LPS did not change upon *in vitro* exposure to IL-1β and TNF-α, as DC-A210 and DC-10 maintained high IL-10 and IL-6 production levels (Figure 5B). Proliferation of T-alloA210 and T-allo10 cells in response to secondary stimulation with allogenic mDC did not change in the presence of IL-1β, IL-6, and TNF-α. Furthermore, T-alloA210 and T-allo10 cell cultures remained anergic in the presence of IL-1β, IL-6, and TNF-α (Figure 5C and data not shown). These data demonstrate that DC-A210 and T-alloA210 cell function is preserved in inflammatory conditions.

## Discussion

In this study, we show that the bi-specific CD86–monomeric IL-10 molecule APVO210 differentiates CD14^+^ monocytes into a subset of DC with tolerogenic phenotype and function. These DC-A210 are CD86^+^ and, unlike mDC, they express CD14 and CD16, but at lower levels compared to tolerogenic DC-10 differentiated in the presence of dimeric human IL-10. In addition, tolerogenic DC-A210 produce high levels of IL-10, and can induce alloantigen-specific anergic T-alloA210 cells that are enriched in CD49b^+^LAG3^+^ Tr1 cells. T-alloA210 secrete high levels of IL-10 and no IL-4, are anergic in response to the alloantigen used during priming, and suppress primary proliferation of autologous CD4^+^ T cells. The phenotype and function acquired by DC-A210 and T-alloA210 cells generated in the presence of APVO210 are comparable to that of DC-10 and T-allo10 generated in the presence of IL-10, and are stable upon exposure to inflammatory cytokines *in vitro*.

Our results demonstrate that delivery of monomeric IL-10 to purified CD14^+^ monocytes using APVO210, a molecule that contains an anti-CD86 antibody, is sufficient to induce tolerogenic DC. The originality of this approach consists in the targeted delivery of IL-10 to the CD86^+^ APC that modulates their antigen-presenting capacity, and therefore their interaction with T cells, without triggering IL-10R signaling in T and B lymphocytes (Hernandez-Hoyos et al., unpublished data). DC-A210 have a stable tolerogenic phenotype and express HLA-G, a non-classical HLA molecule that has immunomodulatory properties and is essential for Tr1 cell induction (33, 58, 59). Indeed, we show that DC-A210 are able to induce alloantigen-specific T cells enriched in CD49b^+^LAG3^+^ Tr1 cells. These T cells produce high levels of IL-10, and are able to suppress primary proliferative T-cell responses.

These findings demonstrate the potent immunomodulatory effect of APVO210 and expand previous data obtained by Aptevo Therapeutics showing that APVO210 is able to inhibit APC function and T-cell proliferation in a primary mixed lymphocyte reaction (unpublished data). The inhibitory effect of APVO210 on primary T-cell proliferation is stronger than that obtained with equal concentrations of a combination of soluble anti-CD86 antibody and soluble monomeric IL-10. APVO210 is also able to inhibit expansion of human T cells in a murine model of xeno-GvHD (see text footnote 1). Our findings support the hypothesis that the *in vivo* protective effect of APVO210 is mediated through the induction of tolerogenic DC and Tr1 cells.

CD86 blockade has been explored in the clinic using different molecules that mimic CTLA4, a natural ligand for CD80 and CD86 that blocks CD28 activating signaling. One of these molecules, abatacept, is an effective therapy in Th1-mediated autoimmune diseases, such as RA (60, 61). However, abatacept is not efficacious for the treatment of Th-17-mediated autoimmune diseases, such as IBD (62) and of Th2-mediated inflammatory or autoimmune diseases, such as asthma (63) and lupus, respectively (64). In addition, pre-clinical studies performed in non-human primates showed no efficacy of using abatacept to prevent transplant rejection. An analog of abatacept with higher affinity for CD80 and CD86 named belatacept was successfully used in phase III clinical trials for kidney transplant recipients, and was approved by the US Food and Drug Administration as therapeutic molecule for this indication in 2011 (65–67). These results demonstrate the efficacy of blocking costimulation and T-cell activation. However, the long-term effect of this approach remains to be determined. In the current studies, APVO210 may harness the effects of costimulatory blockade and IL-10R stimulation. Therefore, the *in vivo* use of APVO210 could be advantageous to achieve long-lasting immunomodulation and operational tolerance *via* the induction of Tr1 cells.

We show that APVO210 can replace rhIL-10 to derive tolerogenic DC from CD14^+^ monocytes *in vitro*. Even though DC-A210 express lower levels of CD14 and CD16 as compared to DC-10, we demonstrate that they secrete equal amounts of IL-10 that is critical for Tr1 cell induction. Furthermore, DC-A210 are as potent as the tolerogenic DC-10 to induce anergic, suppressive T cells, that are enriched in CD49b^+^LAG3^+^ Tr1 cells, which can be established for a variety of allergens (34, 68) and alloantigens (29, 69, 70). Supplementation of APVO210 during the DC-A210–CD4^+^ T-cell co-culture is not required for Tr1 cell induction, indicating that the major effect of APVO210 is on DC; in turn, the DC-A210 produce enough IL-10 to tolerize T cells. In addition, we could speculate that APVO210 bound to the surface of DC-A210 triggers signaling through the IL-10R on DC-A210, and that this signaling persists during the T-alloA210 induction *in vitro*.

The specific ability of APVO210 to deliver IL-10 to CD86^+^ cells (see text footnote 1), as compared to IL-10 which has a pleiotropic effect (7, 9), may have significant advantages for *in vivo* use. The systemic administration of IL-10 *in vivo* has indeed been limited by the development of adverse effects that are due to its stimulatory functions on CD8^+^ T cells (9) and B cells (7, 8). Based on the findings that APVO210 selectively targets APC without triggering IL-10R signaling in T or B cells *in vitro* (see text footnote 1), we hypothesize that the *in vivo* use of this molecule could decrease the risk of triggering aspecific T- and B-cell responses associated with IL-10 systemic delivery, and, therefore, lead to more targeted and safe control of undesired inflammatory and autoimmune responses. Administration of APVO210 *in vivo* could be envisaged to modulate autoantigen presentation in autoimmune diseases, prevent flares of autoinflammatory responses, and decrease T-cell autoreactivity, or alloantigen presentation in HSCT or solid organ transplantation. In addition, our data demonstrate that APVO210 induces differentiation of antigen-specific Tr1 cells, thus providing strong indication that the *in vivo* immunomodulatory properties of APVO210 could be conferred by induction of antigen-specific Tr1 cells. Furthermore, our group has previously shown that *in vitro* induced Tr1 cells and *ex vivo* isolated Tr1 cell clones are able to suppress proliferation, cytotoxicity, and IFN-γ production of CD8^+^ T cells (32, 39). We propose that Tr1 cells induced *in vivo* by the action of APVO210 would constantly be activated by the presence of the antigen, thus proliferating and further produce IL-10 locally, and promote the maintenance of a tolerogenic environment.

We show that alloantigen-specific T-alloA210 cells have a preserved ability to produce IFN-γ and proliferate upon polyclonal stimulation. These data are in agreement with our previous findings that IL-10 anergized T cells specific to an alloantigen have a preserved ability to proliferate in response to *C. albicans, T. toxoid*, and CMV (32). Overall, these data suggest that antigen-specific T-alloA210 cells, like T-allo10 cells, could have a preserved ability to mount an effective immune response against pathogens. The functional properties of DC-A210 and T-alloA210 cells do not change when exposed to inflammatory cytokines *in vitro*. This finding is especially relevant for the use of APVO210 in inflammatory diseases, and in HSCT, as the conditioning regimen given to the host prior to HSCT triggers acute inflammatory reactions that favor GvHD (71–73).

To conclude, our data show that APVO210 is a potent inducer of tolerogenic DC, and of alloantigen-specific Tr1 cells that are stable upon inflammatory conditions. APVO210, therefore, holds promise as a therapeutic agent to prevent or control immune mediated and inflammatory diseases and to induce antigen-specific tolerance.

## Ethics Statement

This study was carried out in accordance with the recommendations of the Stanford IRB based on OHRP and FDA regulations and guidance. This project did not require submission to the Stanford IRB, because the specimens were collected for purposes other than the current research, the identifiers for the data or specimens have been replaced with a code, and the research team is prohibited from obtaining the key to the code. All subjects gave written informed consent in accordance with the Declaration of Helsinki.

## Author Contributions

LP, PC performed the experiments, analyzed the data, and wrote the manuscript. SG discussed the results and reviewed the manuscript. GH contributed to design the experiments, discussed the results, and reviewed the manuscript. RB and MGR designed the experiments, critically interpreted the data, and reviewed the manuscript.

## Conflict of Interest Statement

GH is an employee of Aptevo Research and Development LLC which is a wholly owned subsidiary of Aptevo Therapeutics Inc., and has ownership interest in Aptevo stock. LP, PC, SG, RB, and MR have no conflicting financial interests.

## Footnotes

^1^http://aptevotherapeutics.com/apvo210-anti-cd86-x-monoil-10/.
