# Supplementary material for: APVO210: A Bispecific Anti-CD86-IL-10 Fusion Protein (ADAPTIR™) to Induce Antigen-Specific T Regulatory Type 1 Cells
Source: Front Immunol. 2018 May 25;9:881. doi: 10.3389/fimmu.2018.00881 (PMC5980965; doi:10.3389/fimmu.2018.00881)
Supplement: Supplementary file 1 [file presentation_1.PDF]

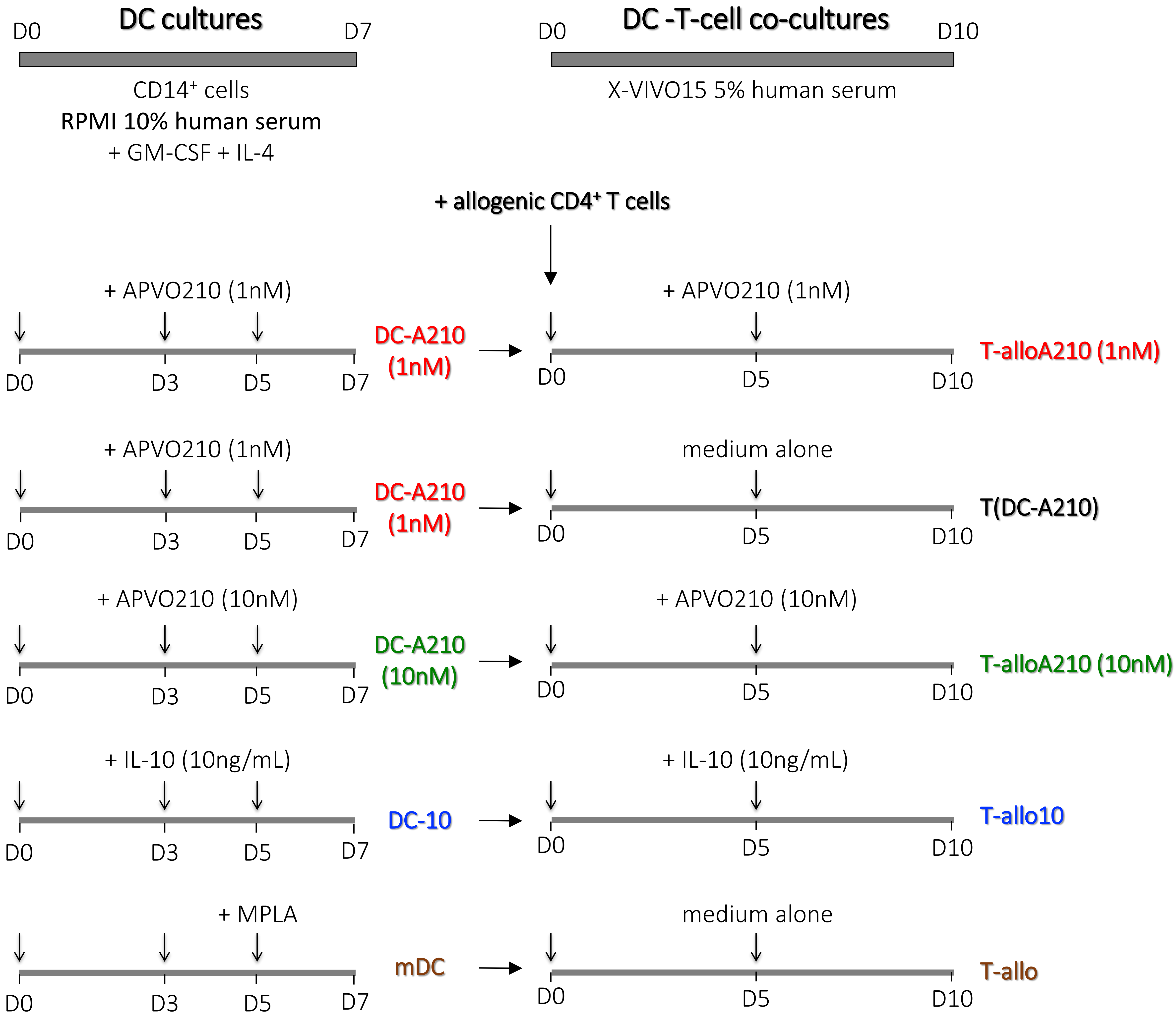

Figure S1. Scheme of the experiments.

DC-A210: DC differentiated in the presence of APVO210; DC-10: tolerogenic DC differentiated in the presence of IL-10; mDC: mature DC. T-alloA210: T cells incubated with allogenic DC-A210. T-allo10: T cells incubated with allogenic DC-10. T-allo: T cells incubated with allogenic mDC.

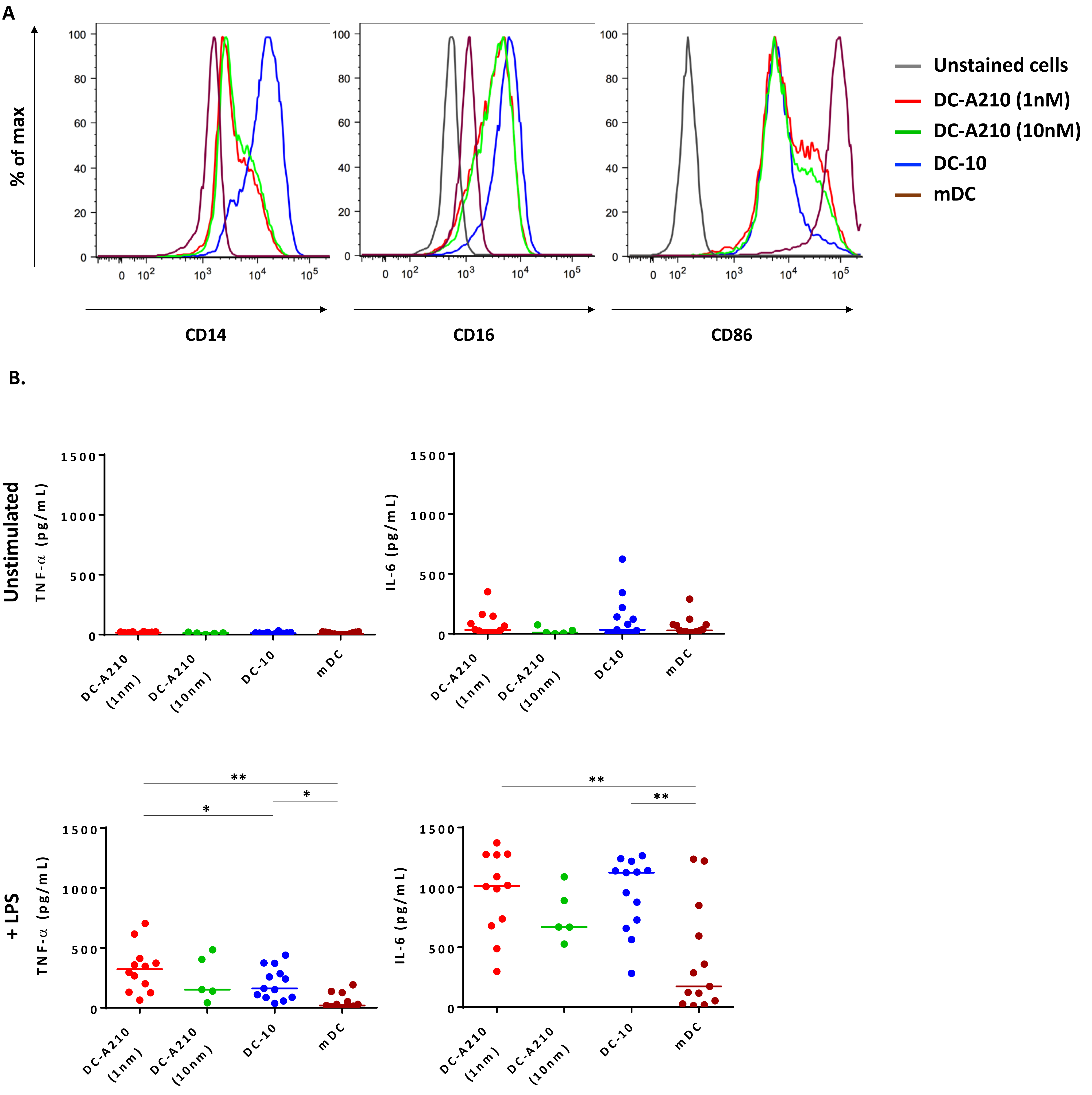

Figure S2. Tolerogenic DC can be differentiated from CD14^{+} monocytes in the presence of APVO210.

DC were differentiated from CD14^{+} monocytes in the presence of IL-4 + GM-CSF (mDC), and of exogenous IL-10 (DC-10), or of APVO210 used at two different concentrations (DC-A210, 1nM or 10nM). **A.** Data of CD14, CD16 and CD86 expression are shown for one representative donor. **B.** Production of TNF-α and IL-6 was evaluated by ELISA in culture supernatants of DC-A210 (1nM, n= 12; 10nM, n=5), DC-10 (n=13) and mDC (n=13) after 48h, in the presence or absence of LPS stimulation. Median values are indicated, each dot represents a single donor and lines indicate median values. Wilcoxon tests with Bonferroni correction were performed for statistical analysis. * $p < 0.05$, ** $p < 0.005$.

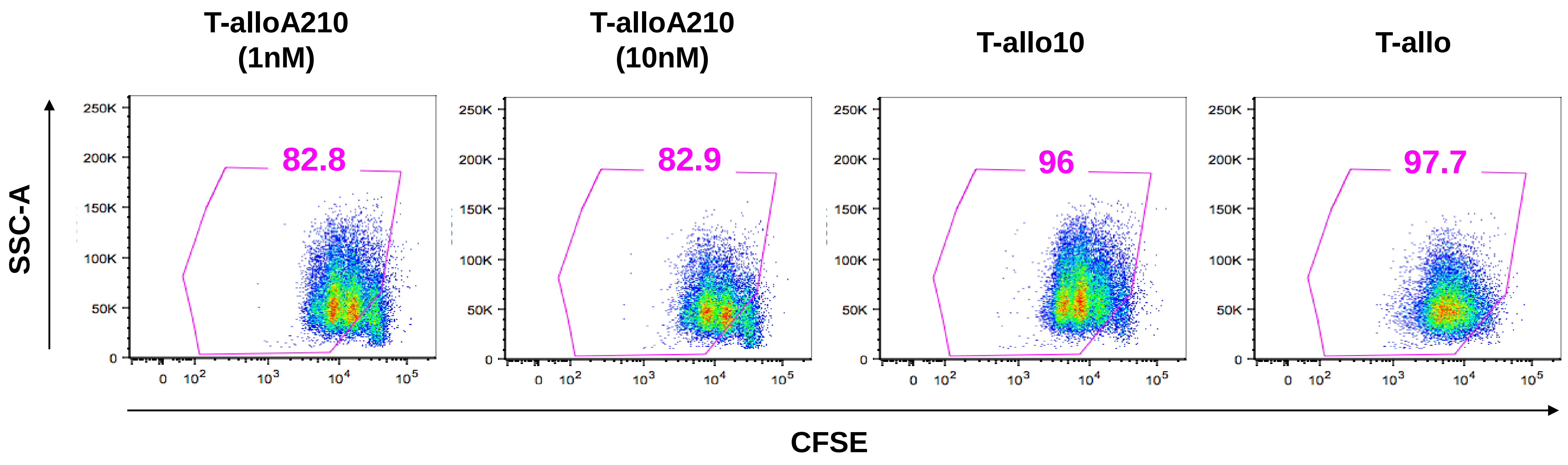

Figure S3. T-alloA210 cells are able to proliferate in response to a polyclonal stimulus.

T-alloA210 (1nM or 10nM), T-allo10, and T-allo cells were stained with CFSE and co-cultured with beads coated with anti-CD3/CD28 antibodies. Proliferation was assessed after 3 days of stimulation by means of CFSE dilution. Data of one representative donor are shown.

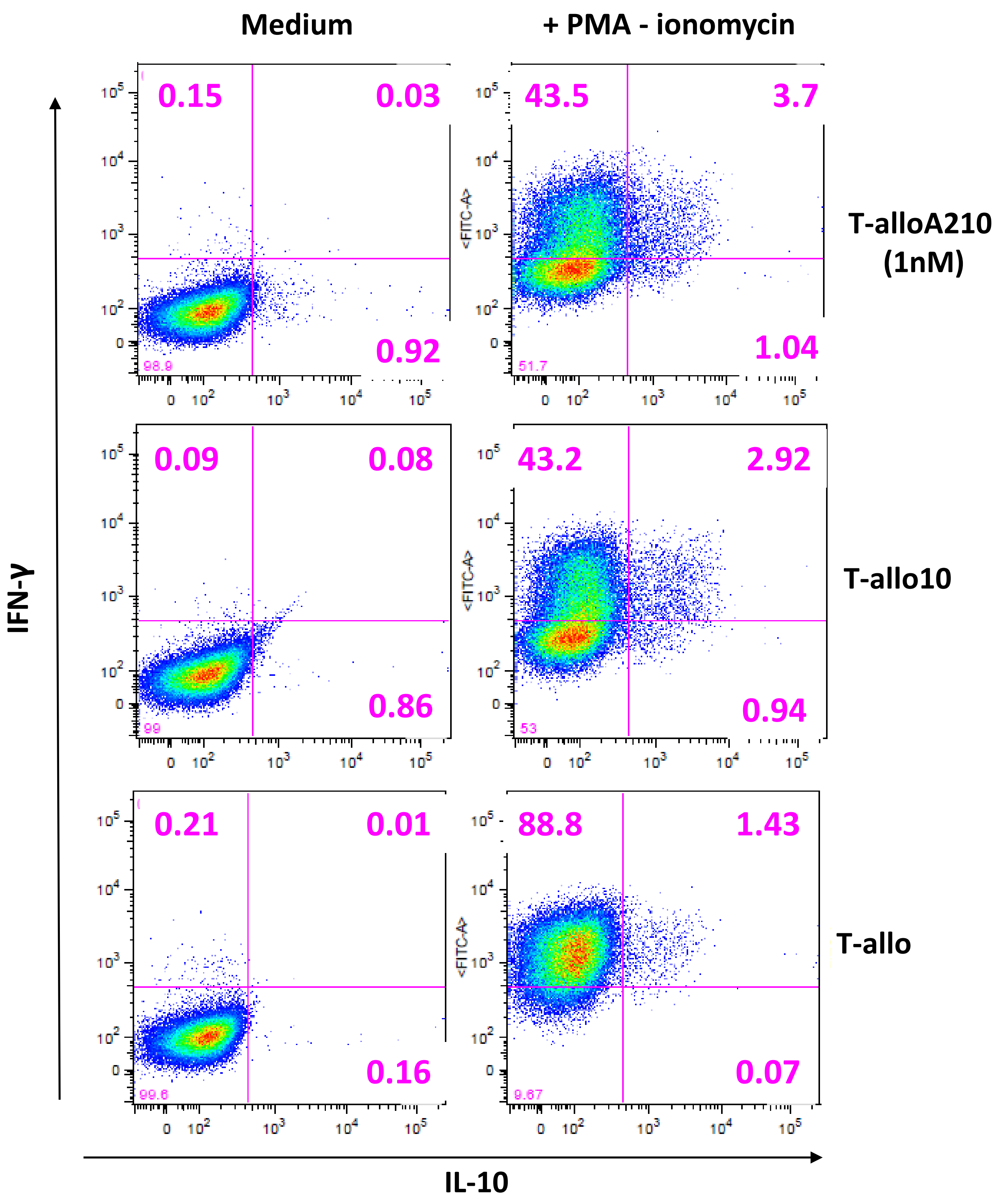

Figure S4. T-alloA210 and T-allo10 cells exhibit a similar cytokine production profile.

T-alloA210, T-allo10 and T-allo were stimulated with PMA and ionomycin for 6h, and percentages of cells producing IL-10 and IFN-γ were evaluated by flow cytometry, using a secretion capture assay. Data of one representative donor are shown.
